# Ginseng leaf-stem: bioactive constituents and pharmacological functions

**DOI:** 10.1186/1749-8546-4-20

**Published:** 2009-10-22

**Authors:** Hongwei Wang, Dacheng Peng, Jingtian Xie

**Affiliations:** 1 Section of Endocrinology, Pritzker School of Medicine, University of Chicago, Chicago, Illinois 60637, USA; 2 Ben May Department for Cancer Research, Pritzker School of Medicine, University of Chicago, Chicago, Illinois 60637, USA

## Abstract

Ginseng root is used more often than other parts such as leaf stem although extracts from ginseng leaf-stem also contain similar active ingredients with pharmacological functions. Ginseng's leaf-stems are more readily available at a lower cost than its root. This article reviews the pharmacological effects of ginseng leaf-stem on some diseases and adverse effects due to excessive consumption. Ginseng leaf-stem extract contains numerous active ingredients, such as ginsenosides, polysaccharides, triterpenoids, flavonoids, volatile oils, polyacetylenic alcohols, peptides, amino acids and fatty acids. The extract contains larger amounts of the same active ingredients than the root. These active ingredients produce multifaceted pharmacological effects on the central nervous system, as well as on the cardiovascular, reproductive and metabolic systems. Ginseng leaf-stem extract also has anti-fatigue, anti-hyperglycemic, anti-obesity, anti-cancer, anti-oxidant and anti-aging properties. In normal use, ginseng leaf-stem extract is quite safe; adverse effects occur only when it is over dosed or is of poor quality. Extracts from ginseng root and leaf-stem have similar multifaceted pharmacological activities (for example central nervous and cardiovascular systems). In terms of costs and source availability, however, ginseng leaf-stem has advantages over its root. Further research will facilitate a wider use of ginseng leaf-stem.

## Background

Ginseng is cultivated in China, Korea, Japan and Russia, as well as in the United States and Canada. Ginseng is one of the most well-known herbal medicines widely used in East Asia as a tonic, restorative and anti-aging agent in traditional Chinese medicine [1-8]. Ginseng is a slow-growing, deciduous, perennial plant of the *Araliaceae *family which includes *Panax ginseng *(*Renshen*, Chinese or Korean ginseng), *Panax japonicus *(Japanese ginseng) and *Panax quinquefolius *(*Xiyangshen*, American ginseng) [9]. Ginseng is used as a dietary supplement in the United States [10].

In Chinese medicine practice, ginseng root is the most commonly used part of the plant. It contains ginsenosides as the major bioactive components known to have complex and multiple pharmacological effects [2,11].

While ginseng leaf-stem was less studied [12], a recent report indicates that American ginseng leaf contains similar pharmacologically active ingredients more abundantly than ginseng root [13]. *Panax ginseng *leaf-stem is rich in containing several ginsenosides. Therefore, this article reviews the constituents and pharmacological profile of ginseng leaf-stem, including its chemical components, biological activities, pharmacological properties and adverse effects.

### Bioactive constituents

Ginseng leaf-stem extract contains a number of important bioactive constituents [14,15], namely ginsenosides, polysaccharides, triterpenoids and flavonoids [16]. Among other constituents, ginsenosides exert main pharmacological actions of ginseng root, leaf-stem and berry [17]. More than 30 ginsenosides have been isolated and identified [10] in *Panax quinquefolius*, *Panax ginseng *and *Panax japonicus *[11,18-20]. Ginsenoside content in the leaf of *Panax quinquefolius *is higher than in the root[21]. However, significant variations in content exist between major ginsenosides in the leaf [13,22-24]. Re and Rd are the major ginsenosides in the ginseng leaf [13,21]. Ginseng leaf-stem may be a valuable source for Re, Rd and Rb2 [23].

Seasonal fluctuations, geographical differences and age variations may affect the ginsenoside content in ginseng leaf. According to a study using solid phase extraction and high performance liquid chromatography (HPLC) on American ginseng leaf and [25], Rh1, Rg2, 20(R)-Rg2 and Rg3 accounted for 4.71% in leaf and 5.35% in berry of American. A RP-HPLC (Reversed-Phase High Performance Liquid Chromatography) study on Rh2 saponin of American ginseng leaf studied the transform ratio of 20(S)-ginsenoside Rh2 [26]. Using HPLC with UV detection at 203 nm, Shi *et al*. found that the seven major ginsenosides (Rg1, Re, Rb1, Rc, Rb2, Rb3 and Rd) were present in various parts of Chinese ginseng of various ages [27]. These results also indicate that ginsenoside content is higher in the leaf and root hair but lower in the stem than that in other parts of the plant and that the total content of ginsenosides in the leaf decreases with age [25-27].

Yan *et al*. developed a simple and reliable liquid chromatography/electron spray ionization mass spectrometry (LC-ESI/MS) assay to detect Chinese ginseng leaf-stem saponin (GLSS) in methanol and rat plasma and to construct the fingerprints of GLSS reference substances and plasma samples. Thirty-one compounds were detected in GLSS, ten of which were identified in the fingerprints of reference substances and the spiked plasma sample. Twelve compounds in GLSS, including C7, C8, C14, C15, C18, Re, C24, Rb(1), Rc, Rb(2), Rb(3) and Rd were easily absorbed and might be the metabolites of GLSS [28]. Moreover, two new compounds were separated from *Panax ginseng *leaf [29].

### Pharmacological functions

Ginseng leaf-stem extracts exhibit multifaceted pharmacological actions in the central nervous system (CNS), cardiovascular system, growth-metabolism system and immune system [30-32] (Table 1). Ginseng leaf-stem extracts also possess anti-fatigue, anti-hyperglycemic, anti-obesity, anti-cancer, anti-oxidant and anti-aging activities as described below.

**Table 1:** Major pharmacological effects of ginseng leaf-stem extracts

**Pharmacological effects**	**Dose**	**Subjects**	**References**
**Central nervous system**			
CNS-depression effects	--	Mice	[30-32]
Anti-electroconvulsive shock	50 mg/kg × 7 days	Rats	[33]
Improving memory	11.25 g/kg *Jiannaoning*	Rats	[36]
**Cardiovascular system**			
Protecting cardiac cell	20 mg/kg iv; 54,27,13.5 mg/kg	Dogs; rats	[39,40]
Preventing coronary vascular dysfunction	120 mg/kg	Rats	[41]
Antagonizing (NE, KCl, CaCl_2_) effects	0.03-3 mg/min	Rabbits; guinea pigs	[42,43]
Anti-CHD effects	--	Patients	[44]
Effects on ANP gene expression	50 mg/kg × 7 d	Rats	[45,46]
**Effects on Growth and metabolism**			
Increasing body weight	--	Young mice and rats	[32]
Effects on lipid metabolism	--	Hyperlipidemic mice	[47]
Regulating lipid metabolism	60 mg/kg	Rabbits	[48]
**Anti-hyperglycemic effects**			
Anti-diabetic effects	--	Diabetic patients	[49]
Anti-hyperglycemic effects	--	Mice, rats	[58]
Lowering blood glucose	150 mg/kg × 12 d	*ob/ob *mice	[21]
Increasing blood insulin	--	Mice; rats	[60]
Hypoglycemic activities	200 mg/kg × 12 d	*ob/ob *mice	[61]
**Anti-obesity activities**			
Decreasing body weight	150 mg/kg × 12 d	*ob/ob *mice	[21,61]
**Anti-cancer effects**			
Anti-prostate, bladder and renal cancer	--	Patients	[63]
Killing cancer cells via at least 5 pathways	--	Normal and cancer cells	[64]
Reducing apoptotic cell number	60-140 mg/kg	Mouse cells	[65]
**Anti-oxidant activity**			
Suppressing antioxidant enzyme activity	40-200 mg/kg	Diabetic rats	[69]
Antioxidant property in cardiac cells	0.25-1 mg/ml	Rat cultured cardiac cells	[8]
Restoring free radical-damaged cells	30 μg/ml (Rb1,2,3)	Cultured myocardiomyocytes	[72]
**Other pharmacological effects**			
Anti-fatigue	100/200 mg/kg	Rats	[73]
Anti-ulcer	100 mg/kg	Mice	[6]
Anti-diuretic	--	Rats	[31]
Anti-aging	--	Patients	[74]
Anti-foot-and-mouth disease	10 μg* +oil emulsion	Mice	[76]

#### Effects on the CNS

An early study revealed that ginseng leaf extract caused CNS depression and neuroleptic effects in mice [30-32]. The extract-induced CNS depression was observed along with a reduction of spontaneous and exploratory movements and the potentiation of hypnotic actions of hexobarbital. Analgesic and anticonvulsant activities were also confirmed in this study. Moreover, ginseng leaf extract inhibited conditioned avoidance response in the pole climbing test.

Effects of saponins from Chinese ginseng leaf-stem on memory, learning and biogenic monoamines of the brain were also examined in rats [33]. Results showed that ginseng root saponins improved learning and memory in normal male rats, while the effects of ginseng leaf-stem saponins on anti-electroconvulsive shock-induced impairment of memory consolidation were more intense. Both leaf-stem and root saponins raised the levels of biogenic monoamines significantly in the brains of normal rats. In another study, the effects of ginseng leaf-stem saponins on learning and memory of one-way avoidance were evaluated in shuttle-box rats [34]. The data indicated that ginseng leaf-stem saponins facilitated the acquisition of learning and memory and ameliorated scopolamine and cycloheximide amnesia. Effects of ginseng leaf extract on the CNS were also examined in various species of ginseng [35]. For example, Siberian ginseng leaf extract was found to have anti-fatigue, anti-stress and anti-depressive effects. An *In vivo *study revealed that a Chinese herbal formula consisting of ginseng leaf, namely *Jiannaoning*, improved memory function in rats with cerebral ischemia [36] and that *Jiannaoning *regulated the levels of interleukin-2, interleukin-6 and neuropeptide Y in rat brain. Moreover, ginsenosides from ginseng leaf-stem affected the level of glucocorticoid receptor (GR) in brain cytosol in heat-damaged rats [37]. Binding activities of GR in brain, lung and liver cytosols and the expression levels of GR mRNA in brain and liver cytosols were all higher in the ginsenosides-treated groups than the untreated control group. Ginsenosides reduced GR binding activity in viscera which may have induced the expression of GR mRNA. Another study [38], however, indicated that extract from the aboveground part of Chinese ginseng (including ginseng leaf-stem) had a weaker effect or no effect on the animal behavior compared to ginseng root.

#### Effects on cardiovascular system

Ginseng leaf extracts had preservative effects on the cardiac and vascular systems and prevented myocardial ischemia in animal experiments [39]. In anaesthetized open-chest dogs treated with American ginseng leaf extract, the myocardial infarct size, activity of serum creatine kinase (CK), lactate dehydrogenase (LDH), the contents of serum free fatty acid (FFA) and lactoperoxidase (LPO) significantly decreased, whereas the activity of serum superoxide dismutase (SOD) and Gtutathione peroxidase (GSH-Px) significantly increased. At the same time, myocardial blood flow was increased and coronary vascular resistance was decreased. The results indicate that the ginseng leaf extract protected against myocardial ischemia by modifying metabolic dysfunction of FFA, inhibiting oxygen free radical-mediated peroxidation of membrane lipids, enhancing endogenous antioxidase activity and increasing myocardial blood supply. Another study [40] confirmed that ginseng leaf-stem extract protects against acute myocardial infarction (AMI) in rats by promoting angiogenesis in the infracted or ischemic area of myocardium.

A previous study demonstrated that Chinese ginseng leaf-stem extracts had beneficial effects on the preservation of cardiac and coronary vascular functions after cold storage for 12 hours in isolated rat hearts. The extracts increased coronary artery dilation and coronary flow in response to an endothelial-dependent vasodilator (ACh), protected the coronary endothelium, prevented coronary vascular dysfunction induced by reperfusion injury after hypothermic heart preservation and attenuated reperfusion damage of vascular smooth muscle cells [41].

Furthermore, American ginseng leaf-stem saponins were reported to antagonize the effects of norepinephrine (NE), potassium chloride and calcium chloride on the isolated aortic strips of rabbits [42]. The saponins inhibited intracellular and extracellular Ca^2+^-dependent contractions induced by NE in rabbit aortic strips. Another study revealed that American ginseng saponins inhibited the contractility of guinea pig papillary muscle [43]. A randomized controlled trial with double blinding indicated that *Shenshao Tongguan Pian*a, a proprietary Chinese medicine formula containing ginseng leaf-stem extract, effectively treated angina pectoris in coronary heart disease (CHD) with effective rates of 94.7% and 67.0% in the treatment and control groups respectively [44].

Several studies revealed that ginseng leaf-stem extract affected atrial natriuretic peptide (ANP) gene expression in older rats [45,46] and that both ginseng leaf-stem and root extracts increased the ANP mRNA in rats. An *in vivo *study on American ginseng leaf extracts showed that the expression of vascular endothelial growth factor (VEGF) and mean micro-vessel density were higher in the ginseng leaf saponin groups than in the vehicle model group and that the expression of basic fibroblast growth factor (bFGF) was higher in the ginseng leaf saponin groups than in the vehicle model group [40], suggesting that ginseng leaf-stem extracts may protect myocardium from ischemic injury in rats with AMI by up-regulating VEGF and bFGF in myocardial cells thereby inducing angiogenesis.

#### Effects on growth and intermediary metabolism

Ginsenosides from Chinese ginseng leaf-stem significantly increased the protein and RNA contents of muscles and liver in rats and that ginsenosides accelerated the growth of young pigs. It was suggested that ginsenosides may have direct influence on RNA and protein synthesis [32].

Ginsenosides from ginseng leaf-stem coupled with aerobic exercise lowered serum lipid, regulated lipid metabolism, promoted antioxidation and enhanced immune activity [47]. Oral administration of ginsenosides extracted from ginseng leaf-stem significantly inhibited the rise of total lipid, cholesterol and triglyceride in rabbits [48].

#### Anti-hyperglycemic effects

One third of diabetic patients use dietary supplements or alternative medicines [49]. Previous studies indicated that ginseng is an important alternative medicine to treat diabetes and both Chinese and American ginseng roots had anti-hyperglycemic effect [50-53]. Ginseng berry extract reduced hyperglycemia and body weight in C57BL/6J *ob/ob *mice [54,55] and C57BL/Ks *db/db *mice [56]. Ginseng leaf-stem extracts also had this anti-diabetic effect [21,57,58]. Ginseng leaf and root extracts increased the basal content and glucose-dependent secretion of insulin in blood [59].

Active ingredients and hypoglycemic properties of American ginseng leaf were examined with high performance liquid chromatography (HPLC) in diabetic *ob/ob *mice [21]. The results indicated that American ginseng leaf extract significantly reduced blood glucose levels. Intraperitoneal glucose tolerance test showed that the leaf extract significantly improved glucose disposal. Thus, American ginseng leaf extract, with its high ginsenoside yield, may be an inexpensive alternative to the root for diabetic treatment. Similar anti-hyperglycemic activity was observed in a study on total ginsenosides of Chinese ginseng leaf-stem [60].

#### Anti-obesity effect

Obesity is a serious medical disorder that may cause a myriad of health problems, such as heart disease, hypertension and adult-onset diabetes. Berry, root and leaf extracts of American and Chinese ginseng as well as total ginsenosides of Chinese ginseng leaf-stem had anti-obesity activities in animals and that American ginseng leaf extract significantly reduced body weight in adult *ob/ob *mice [21,54,56,60,61].

#### Anti-cancer effect

Anti-cancer effect of ginseng leaf-stem is an important pharmacological function. Anti-cancer effects of Chinese ginseng leaf extract were found after co-administration of acidic polysaccharide from Chinese ginseng leaf enhanced therapeutic effects and reduced hematopoietic complications induced by systemic chemotherapy or radiation therapy [62]. Acidic polysaccharide may be a novel and potent immunotropic agent to improve cellular immunity and an anti-cancer drug to treat urological cancer patients. Extract of Indian ginseng leaf (*Ashwagandha*) had anti-cancer activities [63]. Total saponins from *Panax ginseng *leaf-stem protected against cyclophosphamide (a commonly used anti-cancer compound)-induced genotoxicity and apopotosis in bone marrow cells and peripheral lymphocytes in mice [64]. Thus, ginseng leaf extracts can be a new source for anti-cancer drugs.

Kitts *et al*. also confirmed that ginsenoside Rh2 extracted from American ginseng leaf induced cytotoxity in cultured leukemia THP-1 cells [65]. Flow cytometry of cells stained with annexin V-fluorescein isothiocyanate and propidium iodide showed that the Rh2 from ginseng leaf significantly increased apoptosis at a concentration that inhibited cell viability by 50% (LC_50_). Ginsenoside (Rh2) may be the active ingredient for anti-cancer activity in ginseng leaf [65].

#### Anti-oxidant activities

Extracts from American ginseng root and berry possess antioxidant properties [5,13,35,66,67] and so does ginseng leaf extract. In streptozotocin-induced diabetic rats, oral administration of wild ginseng leaf extract (WGLE) effectively suppressed lipid peroxidationin diabetic rats [68]. Similar antioxidant activities were observed in cultivated and wild Korean ginseng leaf extracts [69]. It was confirmed that water, methanol and ethanol extracts form freeze-dried leaves of wild ginseng exhibited scavenging activities towards DPPH (2,2-diphenyl-2-picrylhydrazyl hydrate), superoxide anion and hydroxyl radicals. Among various solvent used to extract wild ginseng leaves, ethanol yielded the highest DPPH, hydroxyl radical scavenging and ferrous ion chelating activity [70].

Not surprisingly, the saponin extracted from American ginseng leaf-stem (0.25-1 mg/ml) also demonstrated antioxidant properties in cultured rat cardiomyocytes [5]. Moreover, Rb1, Rb2 and Rb3 extracted from *Panax ginseng *leaf-stem restored the action potentials of free radical damaged cells [71].

#### Other effects

##### Anti-fatigue effect

Chinese ginseng leaf-stem extract had anti-fatigue effects. In rats, orally administered saponins extracted from Chinese ginseng leaf-stem significantly prolonged swimming time, inhibited the increase of blood lactic acid and reduced liver and rectus femurs muscle glycogen. Ginseng leaf-stem extract also facilitated the synthesis of protein and expression of mRNA in liver and muscle tissues [72].

##### Anti-ulcer effect

*Panax ginseng *root is used in Chinese medicine to treat gastrointestinal disorders. Research showed that the crude polysaccharide fraction from ginseng leaf exhibited potent anti-ulcer activity against acute gastric lesions in mice [3].

##### Anti-diuretic effect

Anti-diuresis was another pharmacological property of ginsenoside from *Panax ginseng *leaf-stem. Total ginsenosides from the leaf-stem helped retain water and Na^+^, increased K^+ ^excretion and reduced the ratio of urinary Na^+^/K^+ ^in rats [73].

##### Anti-aging effects

A clinical trial showed that *Tongbu *No.1, a proprietary Chinese medicine formula containing ginseng leaf, improved various some symptoms related to aging, improved immune and endocrinal functions, scavenged free radicals and adjusted intestinal flora [74].

##### Inductive differentiation effect

Ginsenosides from *Panax ginseng *leaf-stem induced the differentiation of all types of acute nonlymphocytic leukemia cells in primary culture [75].

Saponins, as well as the combination of saponins and oil, significantly enhanced the immune response in mice to vaccination against foot-and-mouth disease (FMDV) [76]. Co-administered with the saponins, FMDV antigen induced a significantly higher IgG response than FMDV antigen used alone.

### Potential adverse effects

Both animal experiments and clinical trials have shown that normal use of ginseng is safe [50,76]. Asian ginseng is classified as a generally safe herb along with feverfew, garlic, ginkgo, saw palmetto, St. John's wort and valerian [77]. Ginseng, including ginseng root and leaf-stem, may exhibit minor adverse effects [78]. A systematic review of adverse effects of ginseng suggested that *Panax ginseng *monopreparations are rarely associated with adverse events or drug interactions [79]. The adverse effects in clinical trials or toxic effects in animal experiments were attributed to improper use or poor quality of ginseng [80-83].

#### Acute toxic effects

Toxicity of ginsenosides from *Panax ginseng *leaf-stem was determined in mice. When mice were given ginsenosides per oral, no death occurred [31,32]. Another report [84] indicated that the LD_50 _values of crude saponin fraction and saponins of ginseng leaves were 381 mg/kg and 299 mg/kg respectively.

#### Subacute toxic effects

Ginseng leaf-stem extracts did not affect the number of erythrocytes, leukocytes, thrombocytes, the amount of hemoglobin or renal function in subacute toxic experiments in rats [32]. Body weight, food consumption and liver weight of rats increased [32]. Brain, heart, lungs, liver, spleen, kidneys, stomach, testes and ovaries were normal on gross examination and histopathological study. These findings suggested that high quality ginseng leaf-stem and its preparations were safe in normal use.

### Limitations of current research

(1) Most studies on the constituents of ginseng leaf-stem extract have been qualitative. Quantitative studies will be required. (2) Quality control of ginseng leaf-stem preparations has not been adequately assured. (3) Few randomized, double-blind, placebo-controlled clinical trials on ginseng leaf-stem extracts are available. Further laboratory and clinical studies are warranted for wider pharmaceutical use of ginseng leaf-stem.

## Conclusion

Extracts from ginseng root and leaf-stem have similar multifaceted pharmacological activities (e.g. CNS and cardiovascular system). In terms of costs and source availability, ginseng leaf-stem has advantages over its root. Further research will facilitate a wider use of ginseng leaf-stem.

## Abbreviations

AMI: acute myocardial infarction; ANP: atrial natriuretic peptide; bFGF: basic fibroblast growth factor; CHD: coronary heart disease; CK: creatine kinase; CNS: central nervous system; CHD: coronary heart disease; FFA: free fatty acid; GLSS: ginseng leaf-stem saponin; GSH-Px: Gtutathione peroxidase; GR: glucocorticoid receptor; HPLC: high performance liquid chromatography; LC/ESI-MS: liquid chromatography/electron spray ionization mass spectrometry; LD_50_: lethal dose to 50% of the sample; LDH: lactate dehydrogenase; LDL: low-density lipoprotein; LPO: lactoperoxidase; NE: norepinephrine; RP-HPLC: reversed phase-high performance Liquid chromatographic; SOD: superoxide dismutase; TCM: traditional Chinese medicine; UV: ultra-violet; VEGF: vascular endothelial growth factor; WGLE: wild ginseng leaf extract.

## Competing interests

The authors declare that they have no competing interests.

## Authors' contributions

HWW, DCP and JTX conceived the topic, collected data and drafted the manuscript. All authors read and approved the final version of the manuscript.
